# Biological, Psychosocial, and Microbial Determinants of Childhood-Onset Obsessive–Compulsive Disorder: A Narrative Review

**DOI:** 10.3390/children12081063

**Published:** 2025-08-13

**Authors:** Alejandro Borrego-Ruiz, Juan J. Borrego

**Affiliations:** 1 Departamento de Psicología Social y de las Organizaciones, Universidad Nacional de Educación a Distancia (UNED), 28040 Madrid, Spain; 2 Departamento de Microbiología, Universidad de Málaga, 29071 Málaga, Spain; jjborrego@uma.es

**Keywords:** obsessive–compulsive disorder, obsessive–compulsive symptoms, etiology, biological factors, psychosocial factors, gut microbiome, gut–brain axis, psychobiotics

## Abstract

The etiology of obsessive–compulsive disorder (OCD) remains incompletely understood, but it is widely recognized as the result of a complex interplay among multiple contributing mechanisms, often emerging during childhood. This narrative review synthesizes current evidence on the etiology of childhood-onset OCD, with particular focus on whether GM alterations are involved in the pathophysiological mechanisms underlying the disorder. Specifically, the review first examines both biological and psychosocial determinants of OCD, and then explores the role of the gut microbiome (GM), including the potential of psychobiotics as a novel therapeutic approach. OCD has a strong hereditary component, involving both common polygenic variants and rare mutations. Epigenetic mechanisms such as DNA methylation and microRNA play a role in mediating gene–environment interactions and influencing OCD risk. Dysfunction and hyperactivity within cortico-striato-thalamo-cortical circuits underlie one of the neurobiological bases of OCD. Infections and autoimmune reactions can trigger or exacerbate OCD, particularly in pediatric populations. A range of psychosocial factors have been implicated in the onset of OCD, often in interaction with underlying neurobiological vulnerabilities. Current evidence indicates that GM alterations may also contribute to OCD pathophysiology through immune-mediated neuroinflammation, disrupted gut–brain signaling, and neurotransmitter imbalance. Individuals with OCD present reduced microbial diversity and lower abundance of butyrate-producing taxa, as well as altered IgA levels and increased infection susceptibility. These shifts may affect dopaminergic, glutamatergic, and serotonergic pathways, particularly via tryptophan metabolism and compromised gut integrity. Thus, the GM plays a pivotal role in OCD, constituting a promising approach for understanding its etiology and highlighting the significant clinical potential of microbial-based treatments such as psychobiotics. Nevertheless, despite progress, gaps remain in understanding childhood-onset OCD determinants, including limited longitudinal studies, incomplete characterization of the GM, scarce psychobiotic trials, and a need for integrated multidisciplinary approaches. Moreover, epidemiological evidence is compromised by reliance on lay diagnoses, questionable assessment validity, and insufficient distinction from related disorders.

## 1. Introduction

Obsessive–compulsive disorder (OCD) is a mental health condition characterized by obsessions, defined as intrusive, unwanted, and persistent thoughts, urges, or images that provoke significant distress, as well as by compulsions, which are repetitive behaviors or mental acts performed to alleviate obsession-related distress, despite being disproportionate or not realistically connected to the feared outcomes [1]. The disorder typically manifests through symptom dimensions such as contamination fears accompanied by excessive washing, fears of causing harm associated with repeated checking, and concerns with symmetry that result in compulsive ordering or arranging. Additional symptoms may include scrupulosity and avoidance behaviors, which can significantly impact daily activities [2].

Obsessive–compulsive symptoms (OCS) can markedly impair the quality of life of those affected and lead to significant functional difficulties, often following a chronic course that does not respond effectively to treatment [3]. Regardless of its primary symptomatology, OCD may involve attentional biases and challenges in performing introspective tasks [4]. Furthermore, research has identified a correlation between OCD and certain psychological characteristics, including but not limited to: dysfunctional beliefs, a perceived compulsion to exercise excessive control over one’s thoughts and immediate surroundings, an amplified sense of responsibility, a perfectionist personality, a high intolerance for uncertainty and the possibility of error, and a pronounced tendency to overestimate potential threats, often of a baseless nature [4,5]. Despite the need to exert control, predominantly over their own thoughts, individuals with OCD employ suboptimal strategies for that purpose, which intensify the manifestation and persistence of symptoms associated with the disorder. These suboptimal strategies include neutralization through mental or behavioral acts, conscious suppression of intrusive cognitions, constant worry, and rumination [5,6].

Pediatric OCD is frequently underdiagnosed [7]. Research indicates that the average age of symptom onset in affected children is around 10 years, with a higher prevalence observed in males. However, this gender difference diminishes by late adolescence [8,9]. A survey of US adults reported that approximately 25% of males with OCD experienced symptom onset before age 10, compared to less than 5% of females [10]. Notably, no gender differences in symptom presentation are observed in later adolescence, and more than half of individuals with OCD report symptom onset prior to 18 years of age [10,11].

From a phenomenological perspective, the severity of OCS exhibits similar patterns across the lifespan [8]. However, differences in the prevalence of specific symptom dimensions emerge when comparing pediatric and adult cases. For instance, children and adolescents tend to report higher rates of harm-related obsessions compared to adults, while religious obsessions appear more frequently in adolescents than in children or adults [8]. Older children and adolescents show increased tendencies toward magical thinking and somatic obsessions compared to younger children [12]. Adolescents display features characteristic of both pediatric and adult OCD presentations, with some studies indicating that sexual obsessions are less common in children than in adolescents and adults [8,12], whereas others report no significant differences between children and adolescents in these symptoms [13]. In this respect, further research is required to clarify how cultural and developmental factors shape the onset and expression of OCS [14].

A substantial proportion of pediatric individuals with OCD present with comorbid psychiatric disorders. A recent meta-analysis spanning the lifespan reported that 69% of individuals with OCD had at least one additional diagnosis, with anxiety disorders, mood disorders, neurodevelopmental disorders, and other related disorders being the most prevalent [15]. In this context, a study focused on children and adolescents found that 74% met criteria for at least one comorbid condition, most commonly generalized anxiety disorder (GAD), major depressive disorder (MDD), attention-deficit/hyperactivity disorder (ADHD), and disruptive behavior disorders such as oppositional defiant disorder and conduct disorder [16]. Comorbidity patterns appear to shift with age; anxiety disorders predominate in pediatric OCD cases, whereas mood disorders are more common in adults [15]. This trend corresponds with evidence showing low prevalence of comorbid depression in early childhood OCD, which increases during adolescence to rates comparable to adults [8,12]. Moreover, children and adolescents with OCD exhibit higher rates of specific phobias and tic disorders compared to adult populations [13].

The etiology of OCD remains incompletely understood, but it is widely recognized as the result of a complex interplay among multiple contributing mechanisms, often emerging during early developmental stages, particularly childhood [17]. These etiological mechanisms include: (i) genetic factors, with individuals having a family history of OCD exhibiting a higher likelihood of developing the disorder [18,19]; (ii) epigenetic factors, involving prenatal and perinatal exposures to adverse conditions such as infections or toxic substances that may alter gene expression and contribute to OCD vulnerability [20,21]; (iii) neurobiological factors, involving dysregulation in key neurotransmitter systems, particularly serotonin, dopamine, and glutamate, as well structural and functional anomalies in brain regions such as the orbitofrontal cortex, caudate nucleus, and the cortico-striato-thalamo-cortical (CSTC) circuitry [22,23,24]; (iv) immunological factors, with immune dysregulation proposed as a potential contributor to the emergence or exacerbation of OCS [25]; and (v) psychosocial factors, with vulnerability to OCD shaped by dysfunctional cognitive patterns (e.g., inflated responsibility, threat overestimation), dispositional traits (e.g., cognitive rigidity, perfectionism, neuroticism), and exposure to stress-inducing life events [23,25,26,27].

Recent advancements in DNA sequencing technologies have enabled the investigation of the human microbiomes, with the gut microbiome (GM) being of particular significance. The GM is part of the gut–brain axis (GBA) and plays a pivotal role in the onset and progression of several mental disorders [28]. A limited number of studies have hypothesized that the GBA may be involved in OCD [29,30], despite evidence pointing to several OCD risk factors that can alter the GM [28,31]. Furthermore, preliminary studies have shown that OCD-like behaviors can be modulated through probiotic treatments in axenic animals [32,33,34]. These findings underscore the importance of further exploring the GBA in OCD, suggesting that the GM could represent not only a contributing factor in its pathophysiology but also a promising target for innovative therapeutic strategies.

Given the multifactorial nature of childhood-onset OCD and the limitations of current treatment approaches, a broader understanding of its etiology is required. Although biological factors have been widely explored, growing interest has turned to the role of the GM as a key modulator in brain development and neuropsychiatric conditions. The bidirectional communication of the GBA and its influence on neurotransmitter systems, immune regulation, and stress responses suggest that the GM may represent an underexplored yet critical component in the pathophysiology of OCD. Moreover, psychosocial factors such as early-life stress (ELS) and certain environmental exposures are known to modulate both brain function and microbial composition, further supporting the need for an integrative framework. This narrative review synthesizes current evidence on the etiology of childhood-onset OCD, with particular focus on whether GM alterations are involved in the pathophysiological mechanisms underlying the disorder. Specifically, the review first examines both biological and psychosocial determinants of OCD, and then explores the role of the GM, including the potential of psychobiotics as a novel therapeutic approach.

## 2. Method

To synthesize the existing literature on the central topic of this review, which is the etiology of childhood-onset OCD with a focus on GM involvement, a non-systematic, narrative approach has been employed [35]. A literature search was conducted across PubMed, Scopus, and Web of Science databases using an iterative strategy with several keywords related to the topic under investigation. The keywords used included conceptual terms such as “obsessive–compulsive disorder”, “childhood”, “onset”, “etiology”, “biological factors”, “neurobiological factors”, “genetic factors”, “epigenetic factors”, “immune factors”, “psychosocial factors”, “psychological processes”, “environmental factors”, “microbial factors”, “gut microbiome”, “psychobiotics”, and “therapeutic tools”. Reference lists from relevant reviews and primary research articles were also screened. The search covered articles published between 2010 and 2025 and was performed between February and May 2025. The time frame was set to focus on the most recent evidence, considering the rapid advances in research over the past decade. No language restrictions were applied, although keywords were limited to English. For articles published in languages other than English and Spanish, online translation tools were utilized for consultation. In addition to the date-limited search, earlier publications were also included if deemed sufficiently relevant to significantly contribute to the review. The criteria for deeming relevance included the impact on key concepts and contribution to understanding childhood-onset OCD. Articles were initially screened by title and abstract for relevance. Duplicates and clearly irrelevant studies were excluded. Articles were also excluded if they did not address OCD in relation to its biological or psychosocial etiology, the GM, or the potential role of psychobiotics in treatment. Full texts of the remaining articles were retrieved, and relevant data were extracted for synthesis. The reviewed studies encompassed participants across a developmental span from early childhood to young adulthood (approximately 4 to 26 years), reflecting typical sampling ranges that often extend beyond the strict onset period.

## 3. Biological Determinants in the Etiology of OCD

The predominant biological factors associated with OCD encompass both genetic and neurobiological origins. Research has demonstrated the significance of genetic factors in the development of OCD, with evidence highlighting dysfunction within the CSTC circuitry as a central neurobiological mechanism underlying OCD symptomatology [9].

### 3.1. Genetic Factors

The existing literature underscores a significant hereditary component in OCD susceptibility [36]. Seminal twin studies estimate OCD heritability at approximately 40–50% [21,37]. In a large-scale analysis involving 16,383 Swedish twin pairs, Mataix-Cols et al. [21] reported that additive genetic factors accounted for 47% (95% CI: 42–52%) of the variance in liability to OCS. Furthermore, the same study, which included a total of 24,768 individuals with OCD, demonstrated that familial risk increases with genetic proximity. Notably, first-degree relatives of affected individuals exhibited a 4- to 8-fold higher risk of developing OCD [21].

To advance understanding of the polygenic architecture underlying OCD, molecular approaches such as genome-wide association studies (GWAS) have been employed to identify genetic variants linked to the disorder [38,39]. GWAS entails the comprehensive statistical evaluation of hundreds of thousands of single-nucleotide polymorphisms (SNPs) across large cohorts to detect variants significantly associated with a given phenotype [40]. Stewart et al. [39] analyzed DNA from 1465 OCD cases, 5557 controls, and 400 families, genotyping approximately one million SNPs genome-wide. However, this sample lacked sufficient statistical power, yielding no genome-wide significant loci. The first well-powered OCD GWAS was conducted by Strom et al. [41] in a European ancestry cohort, successfully identifying the first genome-wide significant locus for OCD. Their results aligned with prior evidence of genetic convergence between OCD and commonly comorbid disorders, including Tourette syndrome and anorexia nervosa, thereby highlighting the disorder’s complex, polygenic nature.

GWAS are widely recognized as effective for identifying common genetic variants associated with OCD. However, they are limited in detecting rare variants. Common risk variants, constituting SNPs with a minor allele frequency (MAF) greater than 1%, generally confer modest individual risk increases (e.g., relative risk ~1.1) but can exert substantial cumulative effects. In contrast, rare variants (MAF < 1%) tend to have larger effect sizes (relative risks ranging from 2 to >20) and often possess greater clinical relevance [42]. To explore rare genetic contributions, early genomic investigations employed copy number variant (CNV) analysis, which identifies genomic segments with deletions or duplications relative to the normal diploid state inherited from parental alleles. Deletions correspond to losses of genetic material, while duplications represent gains. McGrath et al. [43] conducted a large CNV study including 1613 OCD cases and 1789 controls, finding no overall increase in CNV burden among cases. Nevertheless, they reported a suggestive enrichment of large neurodevelopmental disorder-associated deletions, particularly at chromosome 16p13.11. Similarly, Mahjani et al. [44] analyzed CNVs in 993 OCD cases and identified potentially pathogenic CNVs within the 16p13.11 locus, including one deletion and two duplications, further implicating this region in OCD risk.

Whole exome sequencing (WES) constitutes another powerful approach for identifying rare genetic risk factors in OCD. WES targets the protein-coding regions (exons) of the genome, which comprise approximately 1–2% of the total genome but are significantly enriched for disease-associated rare variants [45,46]. A particularly informative application of WES is trio analysis, which sequences an affected individual along with their biological parents. Cappi et al. [47] analyzed WES data from 222 OCD trios and demonstrated an enrichment of de novo mutations predicted to impair gene function in probands. Their analysis highlighted two high-confidence risk genes, *CHD8* and *SCUBE1*, each harboring two damaging mutations in unrelated individuals. Halvorsen et al. [48] expanded on these findings by sequencing 1313 OCD cases, including 587 trios, 644 singleton probands, and 41 quartets (two affected siblings and their parents). Their results confirmed an increased burden of rare, predicted loss-of-function variants in OCD cases, particularly within genes intolerant to such mutations. In case–control analyses, *SLITRK5* emerged as the most salient gene associated with OCD risk.

### 3.2. Epigenetic Factors

Despite the high heritability of OCD, GWAS have identified only a single locus reaching genome-wide significance [41]. As previously stated, this limited resultant data may reflect insufficient statistical power to detect common variants with small effect sizes. Alternatively, a portion of the “missing heritability” may be attributable to gene–environment interactions that influence OCD risk [49]. For instance, a systematic review has implicated childhood trauma and other environmental exposures as potential contributors to OCD onset, likely in conjunction with genetic predisposition [50]. Although there is general agreement that environmental influences play a role in OCD pathophysiology, the underlying mechanisms remain incompletely understood [51]. Notably, such influences may operate through epigenetic mechanisms, altering gene expression and co-expression patterns relevant to disease susceptibility [52,53,54].

In human epigenetics, DNA methylation (DNAm) and microRNAs (miRNAs) represent the most extensively investigated modifications [55]. Epigenome-wide association studies (EWAS) have emerged as valuable complements to genetic approaches in the study of psychiatric and neurodegenerative disorders, including Alzheimer’s disease, anorexia nervosa, depression, and schizophrenia [56,57,58,59]. DNAm patterns can be acquired or lost throughout the lifespan and are susceptible to environmental influences, positioning epigenetic modifications as potential biomarkers for gene–environment interactions in OCD pathophysiology [60]. Campos-Martin et al. [51] conducted a two-step EWAS in a sample of 185 individuals with OCD and 199 controls in Germany, identifying 305 differentially methylated CpG sites, 12 of which showed convergence with regions implicated in the sweet-compulsive brain hypothesis. This hypothesis suggests that dysregulated striatal dopaminergic transmission may impair insulin signaling in OCD. Epigenetic regulation has also been observed in genes such as *BTBD3*, *DLGAP2*, *DLG2*, *GABBR1*, *MOG*, *BDNF*, *SLC6A4*, and *LEPR*, which have been implicated in the neurobiological mechanisms underlying OCD [61,62].

Evidence has implicated miRNA dysregulation in the pathophysiology of OCD. Yue et al. [63] reported aberrant plasma levels of miR-132 and miR-134 in individuals with OCD, both of which are known to influence dendritic spine morphology and synapse formation in the cerebral cortex. Similarly, Kandemir et al. [64] identified significant correlations between specific circulating miRNAs and OCD, further supporting a role for post-transcriptional regulatory mechanisms in the disorder. In addition, Muiños-Gimeno et al. [65] examined genetic variants within the 3′ untranslated regions (3′UTRs) of two isoforms of the *NTRK3* gene, which encodes a neurotrophin receptor implicated in anxiety-related phenotypes. Their analysis revealed a significant association between the C allele of rs28521337, which is located within a miR-485-3p binding site in the truncated *NTRK3* isoform, and the hoarding subtype of OCD. Moreover, variants ss102661458 and ss102661460 were found in predicted binding sites for miR-765, miR-509, and miR-128, the latter of which is highly expressed in the brain and involved in synaptic function and neuronal differentiation. Functional assays indicated that these variants significantly altered miRNA-mediated regulation of *NTRK3*, resulting in a partial restoration of gene expression [65].

### 3.3. Neurobiological Factors

Neurobiological models of OCD are grounded in evidence of structural and functional alterations within specific neural circuits in both pediatric and adult populations [66]. These models emphasize the role of dysregulated communication between brain regions involved in cognitive control, emotion regulation, and habit formation. Such dysregulations are thought to underlie the core symptoms of OCD, including intrusive thoughts and compulsive behaviors.

#### 3.3.1. Structural Abnormalities

Direct and indirect pathways within CSTC loops in the prefrontal cortex are known to exert excitatory or inhibitory influences on higher-order cognitive and behavioral processes, thereby supporting adaptive regulatory control [67]. In OCD, a growing body of evidence implicates dysregulation and hyperactivity within specific CSTC circuits, particularly those involving the orbitofrontal cortex, anterior cingulate cortex, and caudate nucleus, as underlying the pathophysiology of intrusive obsessions and compulsive behaviors [9,68]. Furthermore, aberrant activity within CSTC loops involving the dorsolateral prefrontal cortex, dorsal anterior cingulate cortex, basal ganglia, limbic regions, and thalamic nuclei has been identified as a pivotal neurobiological mechanism contributing to OCD symptomatology [69,70].

The caudate nucleus and putamen receive cortical input, which is processed by the basal ganglia and relayed back to the cortex via the thalamus through two principal routes: the excitatory direct pathway and the inhibitory indirect pathway. Dysregulated activity within this circuitry, particularly hyperactivity of the direct pathway, has been implicated in the pathophysiology of OCD [23]. According to Lack and Crowley [71], the orbitofrontal cortex can modulate both pathways, and its overactivation may disrupt the excitatory-inhibitory balance, thereby contributing to the emergence of compulsive behaviors.

Functional magnetic resonance imaging (fMRI) studies have consistently implicated the orbitofrontal and anterior cingulate cortices in the expression of OCS [72,73]. Increased activation has also been observed in the superior and middle temporal gyri, regions involved in biological motion processing. Choi et al. [74] reported a reduction in volume of the anterior superior temporal gyrus, particularly the planum polare, in OCD patients. A large-scale meta-analysis involving 1830 individuals with OCD and 1759 controls revealed reduced hippocampal volumes in the clinical group, which may underlie visuospatial impairments commonly observed in OCD [75]. Gonçalves et al. [76] further identified volumetric abnormalities in frontal (i.e., increased gray matter in the middle frontal gyrus) and subcortical regions (i.e., increased white matter in the pallidum). Temporal–parietal abnormalities have also been associated with symptom severity, including decreased gray matter in the superior parietal lobe and reduced white matter in the angular and superior temporal gyri. These neuroanatomical alterations have been linked to specific OCD features, such as impaired inhibitory control (pallidum, angular gyrus), executive dysfunction (middle frontal gyrus), compulsive checking (superior temporal gyrus), and visuospatial deficits (superior parietal lobe).

Multiple pathophysiological models have implicated dysfunction within CSTC circuits in OCD [77,78,79,80]. Among these, the orbitofrontal striatal model has garnered significant support, emphasizing the role of the orbitofrontal cortex in OCD symptomatology [78]. Expanding on this, Milad and Rauch [79] proposed dysfunction across three distinct CSTC loops: (i) the affective circuit, involved in emotion and reward processing, originating from the anterior cingulate cortex and ventromedial prefrontal cortex, projecting to the nucleus accumbens and thalamus before looping back; (ii) the dorsal cognitive circuit, related to executive functions including working memory, which arises in the dorsolateral prefrontal cortex and connects to the caudate nucleus and thalamus; and (iii) the ventral cognitive circuit, implicated in motor and response inhibition, starting in the anterolateral orbitofrontal cortex and extending through the putamen and thalamus. More recently, OCD has been conceptualized as a disorder of compulsivity, prompting the identification of a sensorimotor circuit originating in premotor cortical areas, projecting to the putamen and thalamus, and returning to the cortex. This circuit is hypothesized to underlie habit-based (automatic stimulus-response) behaviors that contribute to compulsivity [80,81]. Overall, dysfunction across these CSTC circuits, governing emotional and reward processing, executive control, motor inhibition, and habit formation, may foster cognitive and behavioral rigidity, manifesting as inhibitory deficits and driving the clinical presentation of OCD [77].

In addition, several studies have highlighted functional interactions among CSTC circuits. Notably, communication between the affective and sensorimotor circuits is critical for the formation of habitual behaviors and the emergence of compulsivity [80,82]. Earlier CSTC models, however, underestimated the affective contributions of limbic structures such as the amygdala, which is central to fear and anxiety processing [79]. This is supported by strong anatomical evidence demonstrating extensive amygdaloid projections to widespread regions of the striatum, forming cortico-amygdalo-striatal circuits [80,83]. Consequently, limbic regions, including the basolateral amygdala and hippocampus, both extensively connected to the orbitofrontal cortex, have been incorporated into an updated orbitofrontal striatal model, reflecting a more sophisticated understanding of their role in modulating emotional states relevant to OCD [78,80,84].

#### 3.3.2. Dysregulation of Specific Neurotransmitters

Several key neurotransmitter systems within CSTC circuits, including dopamine, glutamate, and serotonin, have been implicated in the pathophysiology of OCD. Early evidence demonstrating the efficacy of serotonin reuptake inhibitors (SRIs) in treating OCS prompted extensive investigation into the serotonergic system. Dopaminergic involvement became apparent following observations that augmenting SRI treatment with dopamine D2 receptor antagonists yielded clinical benefits. More recently, research attention has shifted toward the glutamatergic system, reflecting its emerging relevance in OCD neurobiology [2].

The serotonin system’s involvement in OCD pathophysiology is primarily supported by the therapeutic efficacy of SRIs in this disorder [85]. Some studies have reported altered serotonin and metabolite levels in the cerebrospinal fluid of OCD patients, which tend to normalize following successful treatment, although such findings remain limited and inconsistent [86]. Genetic investigations have identified associations between OCD and variants in serotonin-related genes, including *SLC6A4*, *HTR1B*, *HTR2A*, and *HTR2C*, although these results are variable [87,88]. Furthermore, altered serotonin transporter receptor binding in regions such as the midbrain has been observed in certain studies, but findings lack uniformity [89]. Among serotonin system genes, the polymorphisms *5HTTLPR* and *HTR2A* have been most consistently implicated in OCD susceptibility [90].

Dopamine has been identified as a pivotal mediator of stereotyped behaviors [91] and plays a crucial role in cognitive and affective functions, notably reward processing, which may be disrupted in OCD [92]. Genetic studies have implicated variations in catecholaminergic genes, including the *COMT* gene, as potential contributors to OCD susceptibility [87,88]. Molecular imaging has further revealed alterations in dopaminergic receptor availability, specifically reductions in striatal D2 receptor binding in OCD patients [93]. The role of dopamine within cortical regions in OCD remains an important area for further research. A randomized, double-blind, placebo-controlled trial demonstrated that enhancing cortical dopamine via catechol-o-methyl-transferase (COMT) inhibition with tolcapone significantly ameliorated OCS over two weeks [94]. In addition, adjunctive treatment with antipsychotics targeting subcortical dopamine receptors alongside selective serotonin reuptake inhibitors (SSRIs) may modulate habit-related circuitry and compulsive behaviors, suggesting that these agents could reduce pathological obsessions or magical thinking and consequently prevent maladaptive compulsions such as excessive handwashing [95].

Glutamate, the principal excitatory neurotransmitter within CSTC circuits, has also been implicated in OCD pathophysiology [96]. Glutamatergic projections from the prefrontal cortex to the striatum are integral to CSTC functioning. Evidence from cerebrospinal fluid analyses and magnetic resonance spectroscopy studies has demonstrated altered glutamatergic metabolite levels in OCD patients, supporting the involvement of glutamatergic dysregulation in the disorder [97]. Moreover, genetic studies have linked variants in glutamate-related genes, such as *GRIN2B* and *SLC1A1*, to OCD susceptibility [98,99]. Genome-wide association meta-analyses have further identified glutamatergic system genes, including *GRID2* and *DLGAP1*, as candidates associated with OCD risk [100].

### 3.4. Immune Factors

Current evidence suggests OCS may be associated with Pediatric Autoimmune Neuropsychiatric Disorder Associated with Streptococcal infection (PANDAS), a condition hypothesized to result from Group A beta-hemolytic *Streptococcus pyogenes* infection [101]. It is proposed that post-infectious autoimmune responses contribute to PANDAS pathogenesis [102]. Supporting this, a large Danish population-based cohort study involving 1,067,743 children demonstrated that those with positive streptococcal tests had an increased risk of developing OCD [103]. Moreover, first-degree relatives of OCD patients show a higher susceptibility to streptococcal infections, further implicating immune factors in OCD etiology [104]. More recently, the PANDAS framework has expanded to encompass a broader range of infectious and autoimmune triggers, leading to the introduction of the term pediatric acute-onset neuropsychiatric syndrome (PANS) [105]. Within this framework, additional pathogens such as *Mycoplasma pneumoniae*, *Borrelia burgdorferi*, Borna disease virus, and *Toxoplasma gondii* have been implicated as potential contributors to immune-mediated PANS and related neuropsychiatric disorders, including schizophrenia [106,107,108].

OCD syndromes have also been reported in association with systemic autoimmune diseases [109]. Secondary OCD manifestations have been documented in autoimmune encephalitis [110] and multiple sclerosis [111]. A large-scale Taiwanese cohort study including 63,165 patients with autoimmune disorders and 315,825 matched controls found an increased incidence of OCD among individuals with prior autoimmune conditions, particularly systemic lupus erythematosus, dermatomyositis, and Sjögren’s syndrome [112]. Similarly, a nationwide Swedish study of 30,082 OCD patients identified a significant association with autoimmune diseases, reporting a 43% increased risk of developing any autoimmune disorder in individuals diagnosed with OCD [113].

In summary, immunological abnormalities have been observed in patients with predominantly primary OCD. However, these findings may be confounded by the inclusion of undiagnosed secondary OCD cases within some cohorts. Various genetic, cytokine, and antibody investigations have suggested a potential role of immune mechanisms in OCD pathophysiology [2,114,115]. Several studies report altered levels of pro-inflammatory cytokines in OCD patients [25,116,117], although results remain inconsistent. For instance, a meta-analysis evaluating serum cytokine changes found no significant differences between OCD patients and controls [118].

## 4. Psychosocial Determinants in the Etiology of OCD

A range of psychosocial factors have been implicated in the etiology and progression of OCD, often in interaction with underlying neurobiological vulnerabilities. Among these, ELS and childhood trauma have been identified as relevant contributors, with stress specifically recognized for its significant role both as a triggering and aggravating factor in symptom onset and exacerbation [119]. In addition, family dynamics, cultural context, and broader psychosocial factors have also been shown to influence the development and course of OCD via psychological mechanisms such as maladaptive cognitive and emotional processing [24,120].

### 4.1. Psychological Mechanisms

From a psychodynamic perspective focusing on childhood and adolescence, OCD has been conceptualized as stemming from unresolved unconscious conflicts and structural vulnerabilities, with symptom expression reflecting developmental fixations and rigid defense mechanisms [121]. However, behavioral and cognitive models represent the most widely recognized traditional psychological approaches to understanding OCD [120].

#### 4.1.1. Behavioral and Cognitive Processes

Behavioral models explain that neutral stimuli become associated with distress through classical conditioning, turning into triggers for anxiety. Compulsive behaviors develop as avoidance or escape responses that reduce distress and are maintained via negative reinforcement [71]. Cognitive models propose that obsessions result from misinterpreting common intrusive thoughts as personally significant, threatening, or morally wrong, which causes distress and leads to compulsions or avoidance [71]. These perspectives were later integrated into the cognitive–behavioral model, which explains OCD as emerging from the interaction between maladaptive beliefs and behaviors. Dysfunctional beliefs, including inflated responsibility, intolerance of uncertainty, thought–action fusion (TAF), perfectionism, and overestimation of threat, cause individuals to appraise intrusive thoughts as highly significant or dangerous [24,71]. This appraisal produces intense distress, motivating compulsive behaviors aimed at reducing anxiety. In turn, these compulsions are negatively reinforced by the temporary relief they provide, maintaining and strengthening OCS.

Individuals with OCD exhibit significant impairments in cognitive flexibility, particularly in extra-dimensional set-shifting tasks, which reflect difficulties in adapting behavior to changing contexts [122]. This deficit in behavioral flexibility contributes to the persistence of rigid obsession-compulsion cycles, as individuals struggle to update their responses based on situational demands [123]. Complementing these findings, OCD patients consistently demonstrate motor inhibition deficits that appear independent of stimulus relevance or comorbid depression, whereas cognitive flexibility, measured by the ability to switch responses, is selectively modulated by disorder-relevant stimuli [124]. Moreover, individuals with OCD tend to exhibit more rigid moral reasoning, providing fewer utilitarian responses to impersonal moral dilemmas compared to healthy controls, a pattern linked to their reduced cognitive flexibility. Trait disgust also modulates moral reasoning in OCD differently than in other anxiety disorders, suggesting distinct emotional and cognitive mechanisms underpinning moral decision-making in this population [125]. In addition to these cognitive deficits, cognitive disinhibition has been proposed as a contributing factor in OCD pathogenesis. Negative priming (NP) paradigms have been used as experimental measures of this function, and research employing varied paradigms and controlling for memory confounds has found comparable NP effects between OCD patients and healthy controls, which challenges broad claims of cognitive disinhibition in OCD and suggests that cognitive biases and metacognitive impairments may better account for OCD symptomatology [126]. In addition to these cognitive deficits, feared-self beliefs or negative self-evaluations have been implicated in OCD-related cognitive processes. A study with 463 nonclinical adults found that both OCS and feared-self beliefs predicted higher and more variable levels of doubt in OCD-relevant scenarios such as washing and checking [127]. However, feared-self beliefs did not predict doubt beyond the influence of OCS, suggesting a mediating role. These findings indicate that feared-self beliefs may increase vulnerability to doubt by impairing the recognition of unlikely possibilities, providing further insight into the cognitive mechanisms underlying OCD. Furthermore, elevated cognitive impulsiveness observed in individuals with OCD may contribute to the psychosocial impairments commonly reported in this population by affecting decision-making, self-regulation, and interpersonal functioning. Nevertheless, as this impulsivity does not appear to be familial, environmental and psychosocial factors likely play a crucial role in shaping these cognitive deficits [128]. Thus, the cognitive–behavioral model can explain why individuals with OCD, despite recognizing their compulsions as excessive and egodystonic (i.e., inconsistent with their values and self-image), feel compelled to perform them to alleviate distress rather than to obtain pleasure, thereby perpetuating the cycle of obsessions and compulsions [24,129]. This dynamic reflects a maladaptive coping mechanism and suggests underlying etiological factors related to impaired cognitive control and emotional regulation.

Recent research has also proposed that episodic memory plays a pivotal role in maintaining an adaptive record of goal-directed actions. From a psychological and etiological perspective, impairments in autonoetic consciousness, a core component of episodic memory involving the capacity to mentally re-experience past events, may underlie compulsive checking behaviors by disrupting accurate self-monitoring and memory confidence. Specifically, deficits in autonoetic consciousness could impair the ability to vividly recall whether actions (e.g., locking a door) were completed, thus promoting repetitive checking in individuals with OCD [130]. Moreover, individuals with OCD exhibit working memory deficits and impaired response inhibition. These cognitive control impairments reflect dysfunctional frontoparietal networks, which contribute to the inability to regulate intrusive thoughts and inhibit compulsive behaviors, thereby playing a central role as mechanisms in OCD pathogenesis [131]. While general retrieval-induced forgetting remains intact in OCD, evidence suggests content-specific inhibitory deficits when processing personally salient material. This selective disruption in memory inhibition further exacerbates the persistence of intrusive thoughts, highlighting the interplay between cognitive control deficits and the maintenance of OCS [132].

As previously stated, dysfunctional beliefs, such as inflated responsibility, overestimation of threat, and intolerance of uncertainty, have been identified as central cognitive components in OCD [133,134]. These beliefs often contribute to the persistence and severity of symptoms by fostering heightened anxiety, pathological doubt, and compulsive reassurance seeking. A recent longitudinal study [135] conducted in Turkey found that obsessive beliefs predicted adherence to safety behaviors during the COVID-19 pandemic through the sequential mediation of OCS and pandemic-related distress, underscoring how these cognitive biases extend beyond clinical OCD to influence broader psychological functioning. Furthermore, additional evidence suggests that pretreatment levels of obsessive beliefs, particularly inflated responsibility and threat estimation, are associated with poorer treatment outcomes, whereas reductions in beliefs about thought control and importance predict better recovery trajectories [136]. These findings reinforce the clinical relevance of targeting dysfunctional cognitive appraisals in OCD treatment. Importantly, OCD-related beliefs differ from delusional thinking in their level of conviction, resistance, and attribution to illness, representing a particular spectrum that encompasses non-delusional beliefs, overvalued ideas, and partial insight [137]. In addition, other cognitive processes may also contribute to OCD pathology. For example, patients with OCD often exhibit slowed attentional disengagement from disorder-relevant visual stimuli, reflecting an attentional bias that may hinder flexible cognition and reinforce compulsive patterns [138]. Furthermore, although framing effects in decision-making do not differ significantly between OCD patients and healthy controls, greater indecisiveness among OCD individuals is associated with longer response times and increased susceptibility to framing, pointing to cognitive processing abnormalities that influence symptom expression [139]. Relatedly, a study comparing clinical samples of individuals with GAD and OCD found that while worry, rumination, and symptoms of both disorders were significantly correlated, rumination did not mediate the relationship between worry and OCD, suggesting distinct cognitive mechanisms underpinning these conditions [140].

#### 4.1.2. Metacognitive and Inferential Biases

The metacognitive model of OCD posits that compulsions originate from intrusive thoughts that are misinterpreted as significant or threatening due to an overestimation of their credibility [24,141]. This cognitive bias may extend to memory processes, where diminished confidence in prior actions sustains compulsions [142]. In addition, an inflated sense of responsibility has been identified as a critical factor driving compulsive behaviors [143]. Despite patients often acknowledging the irrational nature of their symptoms (i.e., egodystonia), there is a dissociation between accurate insight and exaggerated behavior, reflecting a metacognitive impairment in which patients maintain explicit knowledge of action-outcome contingencies but persist in excessive, unnecessary behaviors [144,145]. Further research has shown reduced metacognitive sensitivity during decision-making, characterized by lower confidence in choices and elevated decision thresholds [146,147]. Consistent with this framework, Myers et al. [148] examined three core metacognitive domains implicated in OCS: fusion beliefs (i.e., the tendency to conflate thoughts with actions), beliefs about rituals, and stop signals. These authors reported that each domain was significantly associated with symptom severity and, when analyzed sequentially, incrementally explained variance across symptom measures, supporting their collective and distinct contributions to symptomatology. Notably, fusion beliefs and beliefs about rituals remained significant predictors even after controlling for worry and ordinary cognitive beliefs, underscoring the unique role of metacognitive processes beyond traditional cognitive models. Although stop signals contributed additional explanatory power in some symptom measures, their role requires further investigation. Interestingly, constructs commonly implicated in OCD, such as perfectionism, uncertainty, and inflated responsibility, did not emerge as independent predictors once worry and metacognition were accounted for, highlighting metacognitive factors as central to OCD pathology [148].

Individuals with OCD endorse the belief that their intrusive, unwanted thoughts can influence real-world events, a phenomenon referred to as TAF, which comprises two primary forms: Likelihood TAF and Moral TAF [149]. Likelihood TAF reflects the conviction that experiencing a particular intrusive thought increases the probability of a related negative outcome, which can be directed toward oneself or toward others. Moral TAF involves equating the mere presence of an unacceptable thought with the moral culpability of having performed the corresponding action. Experimental evidence indicates that religiosity, especially within certain Christian contexts, may reinforce moral TAF beliefs, thereby exacerbating OCS by amplifying feelings of guilt and inflated responsibility in response to intrusive thoughts [150]. Correspondingly, elevated levels of negative religious coping have been documented among OCD patients, characterized by spiritual conflict, guilt, and doubt. Such maladaptive spiritual beliefs may intensify intrusive thoughts and compulsive behaviors by heightening core pathological elements of OCD, including responsibility, fear, and uncertainty [151]. Although causal relationships remain to be definitively established, these findings align with cognitive and metacognitive models that emphasize dysfunctional beliefs and appraisals as central to OCD symptomatology, underscoring the need to incorporate cultural considerations in etiological frameworks. A recent study involving 65 OCD patients and 45 healthy controls examined how TAF subcomponents predict variability across four OCS domains [152]. The findings revealed differential associations: Likelihood-Other TAF subcomponent positively predicted the presence of unacceptable thoughts, whereas Likelihood-Self TAF was negatively associated with this domain. Notably, none of the TAF subcomponents predicted obsessing or mental neutralizing symptoms. These results suggest that TAF facets contribute differentially to OCS profiles, supporting a dimensional approach to better capture the heterogeneity of dysfunctional beliefs within OCD. TAF is part of a broader constellation of dysfunctional cognitive biases implicated in the persistence of OCS, including inflated responsibility, threat overestimation, and maladaptive coping strategies such as thought suppression and rumination. These cognitive distortions appear to maintain symptomatology independently of neurocognitive deficits [153,154]. Taken together, these findings highlight TAF as a key metacognitive distortion that exacerbates distress related to intrusive thoughts, reinforcing compulsive behaviors and contributing to the chronicity of OCD. Nevertheless, while psychological inflexibility and TAF likelihood appear to act as transdiagnostic contributors to OCD, their explanatory power may be limited, prompting a need to better capture disorder-specific variations [155].

Recent empirical evidence highlights a distinct form of dysfunctional reasoning, termed inverse reasoning or inferential confusion, as a cognitive mechanism specifically associated with OCD. The Inverse Bayesian Account (IBA) model conceptualizes OCD as characterized by this atypical reasoning process in which individuals prioritize internally generated narratives over sensory evidence, leading to persistent doubt and compulsive behavior [156,157]. Specifically, the IBA framework posits that, unlike typical reasoning grounded in direct sensory input and common sense, individuals with OCD engage in inductive inferences that extend beyond immediate evidence. They construct elaborate internal narratives drawing from personal experiences, hearsay, abstract knowledge, and generalized rules. These narratives generate primary obsessional doubts and secondary inferences about potential consequences, which in turn elicit anxiety and compulsive rituals. This reasoning style is characterized by specific cognitive distortions, including inverse inference, distrust of perception, category errors, and reliance on purely hypothetical scenarios. Notably, the model emphasizes that obsessions emerge from the confusion between imagined possibilities and reality, especially when these align with vulnerable self-concepts, thereby driving the core symptomatology of OCD [156,157]. Within this theoretical framework, Wong and Grisham [157] conducted a study involving 187 participants and provided the first task-based empirical evidence demonstrating that inverse reasoning within OCD-relevant contexts significantly predicted OCS, even after controlling for general distress and cognitive beliefs posited by alternative models. A subsequent study [158] reinforced this, confirming the distinctiveness of inverse reasoning as a mechanism specific to OCD in a clinical sample of 75 individuals, further validating the IBA model. More recently, clinical research has expanded on these findings by linking inferential confusion with the feared corrupted self, which constitutes a specific dimension of feared possible selves implicated in OCD pathology. In a sample of 350 clinically diagnosed OCD patients, inferential confusion significantly predicted symptom severity after adjusting for obsessive beliefs, psychological distress, and comorbidities [159]. Importantly, other feared self-dimensions such as the culpable and malformed selves did not mediate this relationship, underscoring the particular relevance of the corrupted self in this cognitive-affective process. These results highlight the critical role of dysfunctional reasoning and maladaptive self-concept in the etiology of OCD and also suggest that effective interventions should target both inferential confusion and the feared corrupted self.

#### 4.1.3. Emotional Dynamics

Emotion regulation difficulties, alexithymia, and maladaptive coping strategies have increasingly been implicated in the etiology and maintenance of OCD. In a clinical adolescent sample of 93 OCD subjects, significantly higher levels of alexithymia, emotion regulation difficulties, and avoidant coping were found compared to 92 controls [160]. Notably, emotion regulation difficulties mediated the link between OCD and the severity of negative coping, independently of alexithymia or coping scores. Similarly, emotion dysregulation has been shown to mediate the relationship between insecure attachment styles and OCS, underscoring the role of attachment-related emotional processing deficits in the disorder’s persistence [161]. Obsessive–compulsive tendencies are further associated with impaired access to experiential emotional states and heightened doubt in emotional awareness, which may reflect a broader difficulty accessing internal states [162]. Meta-analytic findings confirm that early maladaptive schemas related to mistrust, social isolation, defectiveness, and dependence are moderately correlated with OCD, suggesting that these cognitive-emotional patterns contribute to disproportionate negative expectations and low coping efficacy [163]. Complementing these findings, behavioral inhibition during childhood has been associated with increased OCS in adulthood, independent of social anxiety, with this effect moderated by environmental factors such as overprotective parenting [164]. From a neurocognitive standpoint, the active inference hypothesis posits that OCD results from dysregulated precision weighting between prior beliefs and sensory input, where psychosocial factors modulate maladaptive predictive coding through social learning and emotion regulation, thereby influencing the development and maintenance of OCS [165]. In addition, other integrative models have suggested that OCS emerge from core information and motivational conflicts, influenced by psychological trauma and emotion regulation processes, highlighting the potential of therapies focused on anxiety acceptance and modification of foundational assumptions [166]. Overall, these findings highlight the critical role of early emotional and cognitive vulnerabilities in shaping OCD symptomatology, pointing to early-life stressors as key factors in the development and maintenance of the disorder.

### 4.2. Impact of Early-Life Adversity

Exposure to ELS during critical developmental periods significantly increases the risk of psychopathology later in life. ELS disrupts normal brain development, altering both structure and function, with lasting consequences for mental health and contributing to social disparities and cycles of vulnerability, thereby increasing strain on healthcare systems and societal well-being [167]. Exposure to stress has been linked to OCD onset and exacerbation, especially in vulnerable populations [119,168]. Independent of genetic factors, stressful life events (SLEs), especially during adolescence, play a causal role in the worsening of OCS, underscoring their importance as key environmental risk factors in OCD etiology [169]. Meta-analytic evidence indicates that SLEs preceding OCD onset may be associated with female predominance, later onset, and increased mood disorder comorbidity [27]. Interestingly, SLE-related OCD has also been linked to fewer familial cases and symptom profiles dominated by contamination and cleaning rituals, suggesting a distinct clinical subgroup [170]. In this context, familial risk alone has been associated with a lower likelihood of chronicity, but when combined with female gender and early SLE exposure, it seems that the risk of chronicity increases [171].

The relationship between trauma and OCS varies by trauma and symptom subtype, with evidence from both clinical and non-clinical populations indicating that a range of childhood traumas, rather than a single type, is associated with OCD [50,172]. In the context of ELS, adverse childhood experiences (ACEs) have been proposed as precursors to maladaptive appraisals central to OCD, with anxiety, depression, and experiential avoidance mediating symptom severity and dysfunctional beliefs [173]. Notably, betrayal trauma, which refers to harm caused by trusted individuals through neglect or intentional acts, induces intense emotional distress and has been linked to mental contamination, a core feature of OCD characterized by internalized disgust unrelated to physical contaminants [174]. In this respect, catastrophic betrayals such as sexual assault provoke persistent feelings of dirtiness and compulsive washing, underscoring betrayal’s critical role in symptom maintenance and treatment implications. Furthermore, early experiences of frequent criticism, particularly from caregivers, promote a prevention-focused coping style characterized by heightened sensitivity to judgment and dysfunctional beliefs around responsibility and perfectionism [175]. This coping style contributes to the development and maintenance of OCD-related behaviors, which initially aim to prevent criticism-induced harm but often persist habitually, thus acting as ongoing stressors that exacerbate compulsions.

Among ACEs, bullying victimization stands out as a potent source of distress, particularly through the experience of humiliation. Bullying is a widespread social problem that predominantly occurs during childhood and adolescence in school settings, and is associated with severe negative physical and mental health outcomes [176,177,178]. Humiliation, defined as an intense self-conscious emotion arising from an unfair degradation by others that leads to an internalization of self-devaluation [179], is frequent in contexts where bullying dynamics predominate [180]. Such aversive social experiences can produce traumatic interactions that can culminate in severe, long-lasting psychopathologies and severely negative health and behavioral outcomes [180]. Humiliation is closely related to shame [179,181], an emotion that has been implicated in OCD symptomatology. Experimental research shows that individuals with repugnant obsessions experience heightened shame, which drives compulsive and avoidant behaviors more than anxiety does [182]. All these findings suggest that etiological models and treatments for OCD should address self-conscious emotions such as humiliation and shame, as the threat related to a devalued self-representation can produce great deterioration at a personal level, potentially leading to the onset and exacerbation of the disorder.

Childhood trauma also indirectly influences OCS severity via its impact on attachment avoidance and alexithymia, suggesting that disrupted emotional development and relational schemas mediate symptom intensity [183]. For instance, situational stressors related to the recent COVID-19 pandemic have exacerbated OCS, demonstrating how environmental disruptions interact with vulnerability to intensify compulsive behaviors [184]. This may be explained by pandemic-related public health messaging, which potentially intensified contamination-related obsessions due to the heightened focus on hygiene, thereby amplifying compulsive behaviors [185].

Contrary to expectations, insecure attachment and perceived lack of parental support are more common in adolescents with OCD, but do not necessarily reflect traumatic attachment experiences, which appear more relevant to depressive disorders [186]. Similarly, while bullying victimization links to later OCS, genetically informed studies indicate that shared genetic factors explain much of this association, pointing to gene–environment correlations rather than direct causality [187]. In this context, gene–environment interactions, such as *PGRN* gene variations combined with early trauma, critically influence OCS emergence and comorbid depression, highlighting a complex biological and environmental interplay in OCD pathophysiology [188].

### 4.3. Other Major Psychosocial Influences

#### 4.3.1. Internalized Stigma

Internalized stigma plays a significant role in the manifestation and progression of OCD. Misconceptions about OCD contribute to self-stigmatization, which correlates with increased symptom severity and reduced quality of life [189,190]. This internalized stigma may exacerbate maladaptive cognitive and emotional processes implicated in OCD etiology by increasing stress and impairing adaptive coping mechanisms. Furthermore, stigma-related barriers can reduce treatment adherence, limiting the effectiveness of interventions targeting core cognitive–behavioral mechanisms [191]. Although OCD patients often report low external stigma, self-stigmatization remains a critical factor influencing their mental health outcomes [192]. Cultural, socioeconomic, and familial contexts further modulate OCD expression and access to care, especially in ethnic minority populations, where culturally specific stressors, such as racism, reduced mental health resources, and stigma, compound symptom chronicity and impede early intervention [193].

#### 4.3.2. Family Environment and Parenting

Early maladaptive parenting patterns and insecure attachment styles have also been implicated in OCD etiology, potentially fostering deficits in emotion regulation and reinforcing dysfunctional self-beliefs. Individuals with OCD report greater exposure to toxic parental behaviors, including emotional inhibition, shame induction, and unrelenting standards, alongside dismissive attachment patterns and difficulties regulating affect [194]. Maternal overprotection has been shown to predict OCS through the mediation of inflated responsibility attitudes, supporting a developmental-cognitive pathway in OCD [195]. Furthermore, patient perceptions of family criticism and hostility strongly predict symptom severity, with relative-rated hostility emerging as a particularly salient predictor [196]. In this context, family accommodation is prevalent in pediatric OCD and has been recognized as a critical factor influencing the development of the disorder [197,198]. Specifically, it correlates with symptom severity and functional impairment, and also mediates symptom-related disability [198]. Family accommodation refers to behaviors by parents or family members aimed at reducing the child’s distress related to OCS. Such accommodation includes facilitating ritual completion or providing reassurance, both intended to alleviate discomfort and support the child’s well-being. These behaviors are negatively reinforced by decreases in child distress and disruptions to family routines, thereby perpetuating OCD symptomatology. Common forms of accommodation include reassurance provision, participation in rituals, and family-wide avoidance of the child’s feared stimuli [199]. Although accommodation may offer short-term relief, it often has detrimental long-term effects on both the individual and the family system [200]. Elevated family accommodation levels not only correlate with overall increased OCD severity, but also with parent-rated functional impairment and higher frequencies of externalizing and internalizing behaviors [197]. A systematic review further linked greater accommodation with symptom severity and resistance to therapeutic and pharmacological treatments [201]. Despite evidence that accommodation worsens symptoms and treatment outcomes and contributes to parental distress, family members often struggle to resist accommodating behaviors. Requests for accommodation by children, when denied, can provoke anger and, in some cases, abusive reactions [202,203].

#### 4.3.3. Dermatological Health and Psychosocial Interactions

Emerging evidence from psychodermatology highlights the bidirectional relationship between dermatological health and psychiatric disorders such as OCD, suggesting that skin conditions may play a psychological role in the development and exacerbation of certain mental health conditions and vice versa [204,205]. Dermatological diseases such as atopic dermatitis, acne, or psoriasis are not only physically distressing but also provoke a significant psychological distress, often leading to social withdrawal, heightened self-consciousness, and internalized stigma [204]. These psychosocial stressors can act as potential risk factors or maintaining mechanisms for OCD symptomatology, particularly in individuals predisposed to anxiety or compulsive behaviors. Moreover, OCD-relevant behaviors, such as compulsive washing or skin picking (i.e., excoriation), may develop or worsen in the context of visible skin lesions or perceived contamination, illustrating a dynamic interaction between physical appearance and the manifestation of OCS. Thus, given its emotional and social salience, skin health may also represent an underrecognized psychosocial factor in the etiology of OCD. For this reason, its integration into psychiatric evaluations, especially in childhood-onset cases, could inform more comprehensive, biopsychosocial treatment approaches.

#### 4.3.4. Lifestyle Patterns: Physical Activity and Diet

Physical activity typically occurs within social contexts, and dietary habits are strongly influenced by cultural factors, underscoring the inherently social nature of these lifestyle behaviors. Thus, lifestyle factors may also constitute important psychosocial influences on the onset, trajectory, and severity of OCD. Longitudinal evidence shows that lower consumption of vegetables and reduced engagement in moderate physical activity predict worsening OCS over time, while higher intake of high-fat foods correlates with increased compulsive behaviors [206]. Consistent with this, individuals with OCD commonly report various health-related problems and engage in unhealthy behaviors such as physical inactivity, poor diet, risky alcohol consumption, and disrupted sleep quality, which may exacerbate symptoms and act as environmental stressors interacting with genetic and cognitive vulnerabilities to further establish compulsive behaviors [207]. Moreover, OCD is associated with an elevated risk of cardiometabolic disorders, including metabolic syndrome and cardiovascular disease, primarily driven by specific environmental factors rather than familial or genetic influences, as indicated by minimal familial co-aggregation [208]. Interestingly, longer and higher-dose SRI treatment has been linked to reduced risk of these complications, suggesting potential protective effects of pharmacological management on physical health outcomes [209]. Collectively, these findings emphasize lifestyle factors as modifiable psychosocial contributors to the etiology and maintenance of OCD, highlighting the importance of addressing these elements in comprehensive treatment strategies.

#### 4.3.5. Functional Impact and Psychosocial Burden

It is evident that OCD exerts a substantial psychosocial burden. For instance, a large population-based study in Sweden demonstrated that individuals with OCD attain lower educational milestones across the lifespan compared to unaffected siblings, with early-onset OCD associated with greater impairment [210]. These deficits persist even when accounting for psychiatric comorbidities, underscoring the pervasive impact of OCD on academic and occupational trajectories. Such functional impairments may, in turn, increase psychosocial stress and reduce adaptive coping resources, thereby reinforcing OCS chronicity and complicating recovery. Therefore, this bidirectional relationship further highlights the importance of integrating psychosocial outcomes into etiological models to fully understand OCD progression.

## 5. The Gut Microbiome in OCD

The human GM consists of numerous species of microorganisms essential for metabolizing indigestible compounds, producing metabolites, hormones, and vitamins, developing intestinal lymphoid tissue, and preventing colonization by pathogenic microorganisms [211]. The GM comprises both autochthonous and transient microorganisms belonging to the Archaea, Bacteria, and Eukarya domains, as well as viruses and protozoa [28]. The composition of the GM achieves a homeostatic state among all its constituents, resulting in the establishment of complex trophic interactions both within the microbial community and with the human host. However, diverse factors can cause disturbances in this delicate microbial balance, thus altering the homeostasis of the ecosystem and leading to a dysbiotic state [212].

### 5.1. GM Dysbiosis and OCD

Previous studies using animal models suggest that OCD symptomatology could be associated with shifts in the microbial composition of the GM, although there is no direct evidence to demonstrate this relationship. The main microbial pathogens associated with the occurrence of OCS include bacteria, such as *Borrelia*, *Mycoplasma*, and *Streptococcus*; viruses, such as Borna disease virus, Herpes simplex virus 1, *Orthorubulavirus*, and Varicella-zoster; and parasites, such as *T. gondii* [213,214,215,216]. Table 1 presents various preclinical and clinical studies establishing dysbiosis patterns in animal samples and in OCD patients.

Pediatric, neuropsychiatric, and autoimmune disorders linked to PANDAS are marked by the manifestation of OCD-like symptoms [224,225]. A study reported that streptococcal infections altered intestinal bacterial communities with increased inflammation. Patients aged 4–8 years with PANDAS showed increased abundance of the genera *Bacteroides*, *Odoribacter*, and *Oscillospira*, and decreased abundance of the genera *Coprococcus*, *Dorea*, *Roseburia*, and *Turicibacter* [219]. However, very few studies in humans have focused on establishing the connection between GM composition and OCD development. Turna et al. [220] conducted a pilot study involving 21 patients with OCD and found that there was a significantly lower relative abundance of three bacterial genera in their feces: *Anaerostipes*, *Odoribacter*, and *Oscillospira*. These bacteria are major producers of butyrate, a short-chain fatty acid (SCFA) that provides energy and maintains the integrity of the intestinal epithelium, preventing enteric inflammation [226].

Subsequently, Domènech et al. [221] observed an imbalance in the GM in OCD patients, characterized by a decrease in bacterial species diversity and an increase in the genus *Alistipes* (family *Rikenellaceae*), which is related to neuroinflammatory processes, as well as a decrease in members of the families *Prevotellaceae* and *Lachnospiraceae* (genera *Agathobacter* and *Coprococcus*). An association of the genus *Coprococcus* with the synthesis of 3,4-dihydroxyphenylacetic acid, a metabolite of dopamine, has been established. In this regard, dopaminergic transmission plays a critical role in OCD neurobiology, evidenced by the efficacy of dopaminergic receptor agonists as adjunctive treatments to SSRIs in symptom reduction, and by observed increases in striatal dopamine release following deep brain stimulation in OCD patients [227,228]. Furthermore, members of the *Prevotellaceae* family prevent intestinal colonization by microbial pathogens [229].

Recently, Dai et al. [223] conducted a study involving 49 children and adolescents diagnosed with OCD and reported a significantly lower α-diversity in the GM of the OCD group compared to controls. Moreover, distinct microbial biomarkers differentiated the two groups: OCD patients exhibited increased abundance of the Bacteroidota phylum, whereas the Bacillota phylum predominated in controls. At the genus level, *Bacteroides*, *Lachnospira*, *Parabacteroides*, and *Parasutterella* were elevated in OCD, while *Coprococcus*, *Escherichia*, *Mitsuokella*, *Romboutsia*, *Ruminococcus*, *Shigella*, *Subdoligranulum*, and *Terrisporobacter* were more abundant in controls. Notably, several genera reduced in OCD are key butyrate producers, and butyrate plays a pivotal role in central nervous system (CNS) function by modulating hippocampal activity and enhancing BDNF expression, which is associated with anxiolytic and antidepressant effects in animal models [230]. Therefore, diminished butyrate-producing bacteria in OCD patients may lead to reduced SCFA levels, potentially contributing to OCD pathophysiology via inflammatory mechanisms.

He et al. [231], in another recent study, performed a two-sample Mendelian randomization (MR) analysis to investigate the causal relationship between the GM and OCD. Using inverse variance weighting, their results identified the phylum Pseudomonadota, family *Ruminococcaceae*, and genus *Bilophila* as protective factors against OCD. In contrast, the order Bacillales, along with the taxa *Eubacterium ruminantium* group and *Lachnospiraceae* UCG001, were associated with an increased risk of OCD. Importantly, reverse MR analysis did not reveal any significant causal effect of OCD on GM composition, and no evidence of heterogeneity or horizontal pleiotropy was detected.

**Figure 1:**
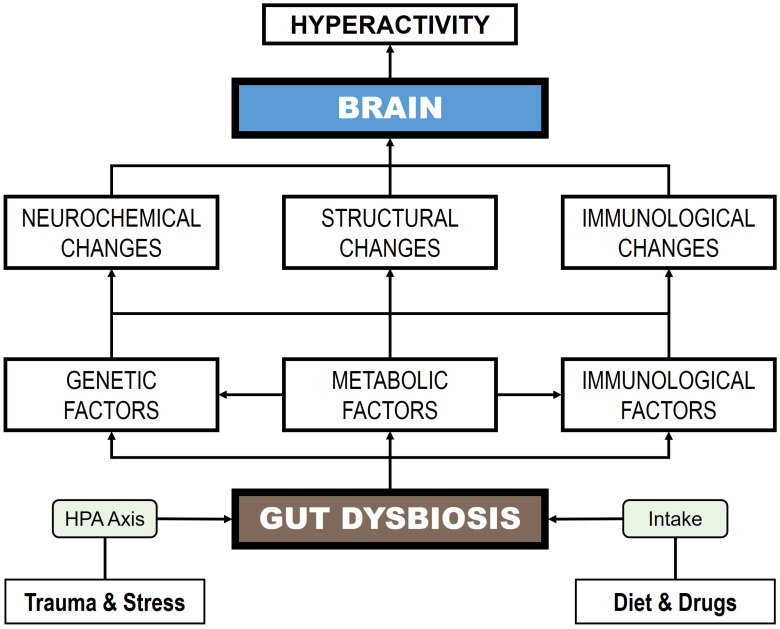
Hypothetical model of the influence of the GM on OCD. HPA: hypothalamic–pituitary–adrenal.

These findings indicate a potential causal association between GM alterations and the emergence of OCD-like behaviors. Nevertheless, further research is essential to fully elucidate the relationship between OCD and the GM, to establish causality, and to clarify its clinical relevance. Figure 1 presents a hypothetical model illustrating how GM dysbiosis may influence multiple etiological factors implicated in OCD (modified from Bendriss et al. [232]).

The developmental processes of the immune system, the GM, and the CNS occur concomitantly during the prenatal period, and present an interdependence among them [233]. Any alteration in microbial composition due to intrinsic or extrinsic factors can lead to irreversible shifts in immune and mental function [234]. It has been posited that some of these shifts may constitute risk factors for the subsequent onset of OCS, establishing a relationship between immune parameters and the GM [235]. Inflammation and immune dysregulation are increasingly recognized in OCD pathophysiology, with preliminary data reporting abnormal IgA levels in pediatric OCD populations. Given the role of IgAs in modulating microbial communities and maintaining mucosal homeostasis through the regulation of bacterial growth and gene expression, these findings suggest a mechanistic link between immune function and microbial dynamics in OCD [236].

A balanced and stable GM (state of eubiosis) provides protection against infections by pathogenic microorganisms through different mechanisms, such as interspecific microbial competition, bacteriocin production, and SCFA synthesis, which preserve epithelial barrier integrity and facilitate immune cell differentiation and the maturation of secondary lymphoid tissues [237,238]. Interestingly, patients with OCD appear to be more susceptible to microbial infections, or to the severity of their symptoms, although it is not known whether these facts are due to infection per se, or are negative neuroimmune consequences, or are due to both factors together [103]. Isung et al. [239] found that IgA immunodeficiency was associated with OCD risk, although it is unclear whether this deficiency was due to self-inflammatory or autoimmune processes or was a consequence of changes in the GM. Shifts in the composition of the GM, including a higher relative abundance of *Eubacterium dolichum* and *Ruminococcus bromii*, and a lower relative abundance of members of the *Paraprevotellaceae* family, have been detected in subjects with selective IgA deficiency [240].

### 5.2. GM Dysbiosis and Neurotransmitters

The hyperactivity observed in OCD is thought to be mediated by dysregulation of key neurotransmitters, including dopamine, glutamate, and serotonin [2,91,96]. Alterations in GM composition can disrupt serotonin synthesis by affecting tryptophan metabolism, a key precursor of serotonin [241]. Although peripheral serotonin does not cross the blood–brain barrier (BBB), tryptophan can cross the BBB via specialized transporters and is subsequently converted to serotonin by neurons within the CNS. GM dysbiosis may reduce tryptophan availability and compromise BBB integrity, facilitating the entry of otherwise excluded molecules into the brain. Certain bacterial taxa metabolize tryptophan, thereby modulating its systemic levels and impacting central serotonin production [242]. In addition, dysbiotic shifts in the microbiota have been linked to altered expression and function of cerebral serotonin receptors, influencing serotonergic signaling pathways. These receptor modifications have been correlated with behavioral phenotypes such as impaired social interaction and heightened anxiety-like behavior [243,244].

One study reported increased dopamine transporter density in the left caudate and left putamen of untreated OCD patients [232]. Antipsychotic medications, often prescribed for OCD cases resistant to SRIs, act by blocking subcortical dopamine receptors and are thought to modulate the habit system and compulsive behaviors. Dysbiosis-driven alterations in GM composition can influence dopamine synthesis and metabolism. Multiple studies have demonstrated that changes in the GM affect dopamine receptor expression and dopamine transporter function. For instance, Bercik et al. [245] showed that germ-free mice lacking gut microbiota exhibited altered dopamine receptor expression in specific brain regions relative to controls with normal microbiota. Furthermore, Dinan et al. [246] conducted a study based on the application of psychobiotics and found that *Bifidobacterium* and *Lactobacillus* species can modulate dopamine receptor levels and dopamine transporter activity [247]. More recently, Hamamah et al. [248] provided evidence that gut microbes, including *Bacteroides*, *Bifidobacterium*, *Clostridium*, *Enterococcus*, *Lactobacillus*, *Prevotella*, and *Ruminococcus*, influence dopaminergic signaling, with dysregulation potentially contributing to dopamine-related pathologies.

A seminal study by Rosenberg et al. [249] demonstrated that untreated OCD patients exhibit elevated glutamate concentrations in the caudate region compared to healthy controls. Notably, this glutamate elevation normalized after 12 weeks of SRI treatment, suggesting that serotonin availability in the frontal regions of CSTC circuits modulates glutamate levels in the caudate. Low serotonin levels may reduce inhibitory control within the circuit, thereby permitting increased glutamatergic activity [250]. The GM influences the glutamate system through two primary mechanisms. First, certain bacterial species, such as *Bifidobacterium* and *Lactobacillus*, are capable of producing and metabolizing glutamate, directly affecting its systemic levels. Dysbiosis-induced alterations in GM composition can thus impact glutamate production and metabolism. Strandwitz et al. [251] identified specific bacterial enzymes that contribute to glutamate synthesis, underscoring the role of the GM in regulating glutamate availability. Second, GM dysbiosis can modulate the expression and function of glutamate receptors and transporters in the brain. For instance, alterations in the GM have been associated with changes in NMDA receptor and EAAT3 transporter expression. Sharon et al. [252] demonstrated that mice with disrupted GM exhibited altered expression of these glutamate-related proteins alongside neurobehavioral abnormalities, highlighting the influence of the GBA on glutamatergic neurotransmission and associated behaviors.

### 5.3. GM Dysbiosis and Environmental Factors

Childhood trauma and ELS can influence the GM composition, potentially triggering dysbiosis [167,253]. In addition, early-life factors such as lifestyle changes and frequent antibiotic use are critical determinants in shaping the developing GM and may contribute to the emergence of neurodevelopmental disorders [233,254,255]. Human studies reveal emerging patterns and gaps in the relationship between ELS and the GM [167]. Prenatal stress is consistently associated with increased Pseudomonadota, particularly *Enterobacteriaceae*, and decreased *Bifidobacterium*, a genus with immunomodulatory and neuroactive properties. These shifts correlate with maternal cortisol and early infant stress markers, suggesting stress-related pathways influencing microbial colonization. Postnatal findings are more inconsistent due to variability in stress definitions, microbiome analysis, and population characteristics. Some studies report reduced alpha-diversity and altered beta-diversity in children exposed to trauma, while others do not, highlighting the need for greater taxonomic resolution and longitudinal designs. Inconsistent findings for *Bacteroides*, *Coprococcus*, and *Eubacterium* linked to both beneficial and adverse outcomes reflect methodological limitations and confounding factors such as sex, medication, diet, and socioeconomic status. Furthermore, genera including *Coprococcus*, *Prevotella*, and *Veillonella* show associations with psychosocial stress across developmental stages, suggesting a potential shared microbial signature [167].

Importantly, GM development and brain maturation occur synchronously from birth through the first three years of life, with the GM playing a pivotal role in processes such as nerve myelination [256]. The GM contains bacteria capable of synthesizing several neurotransmitters, including serotonin (as summarized in Table 1). Disruptions in the GM during these formative years can impair gastrointestinal function and overall health, increasing the risk of various mental health disorders later in life [257]. In addition, early GM dysbiosis may alter physiological trajectories and increase susceptibility to allergic diseases, metabolic disorders, type 1 diabetes, inflammatory bowel disease, and atherosclerosis. Stress-related GM changes may involve epigenetic modifications affecting GBA function. Despite evidence of GM alterations linked to prenatal and postnatal stress, a consistent microbiome signature has not been clearly established [257].

### 5.4. Comparative GM Dysbiosis in OCD and Related Conditions

OCD and autism spectrum disorder (ASD) frequently co-occur, posing significant challenges for clinical management in pediatric populations [258]. Both conditions share characteristic features, including a propensity for ritualistic routines, repetitive behaviors, restricted interests, and resistance to change [259]. Although repetitive behaviors are common to both disorders, their underlying motivations and functional roles differ markedly. In OCD, compulsions are primarily performed to mitigate anxiety or distress stemming from intrusive obsessions. Conversely, in ASD, repetitive behaviors often serve as mechanisms for self-regulation, sensory stimulation, or may reflect an individual’s particular mode of engagement with the environment [260]. Given these symptomatic convergences, several authors have hypothesized a potential etiological and phenomenological relationship between ASD and OCD. The association between these disorders has been widely recognized yet remains contentious. Some researchers maintain that OCD and ASD are frequently comorbid but represent distinct clinical entities, whereas others argue for their conceptualization as points along a shared neurodevelopmental spectrum [261].

ADHD is a neurodevelopmental disorder characterized by persistent patterns of inattention, hyperactivity, and impulsivity, typically emerging in childhood and frequently persisting into adulthood. A substantial body of evidence has documented a high rate of comorbidity between ADHD and OCD in pediatric populations. A meta-analysis of 42 studies involving children and adolescents with a primary diagnosis of OCD reported a pooled prevalence of ADHD comorbidity of approximately 19% [262]. Although ADHD and OCD are clinically distinct entities with separate diagnostic criteria, they share convergent features, particularly in domains of executive functioning, such as deficits in attentional control, planning, decision-making, and impulse regulation [263]. This symptomatic convergence can pose diagnostic challenges, especially in cases where both disorders co-occur [264].

A comprehensive review of the current literature on family and genetic studies indicates a high degree of heritability for both ADHD and OCD. Notably, several genetic variants have been implicated in both disorders, suggesting the possibility of shared pathophysiological mechanisms. Nevertheless, other genetic findings point to distinct etiological pathways, supporting a degree of divergence between ADHD and OCD [265]. Neuropsychological and neuroimaging research has revealed a partial convergence in the executive function deficits associated with each disorder. Specifically, both conditions have been linked to frontostriatal dysfunction, implicating this circuitry in their pathogenesis. Functional brain imaging studies have shown that individuals with ADHD and OCD exhibit both shared and disorder-specific neural activation patterns during tasks involving interference inhibition and attentional allocation. These patterns suggest that differential dopaminergic modulation of striatal regions may underlie some of the observed differences in cognitive control processes. The majority of activation deficits in both disorders have been localized to the frontostriatal, insular, and cerebellar regions, which constitute areas that are implicated in self-regulation, temporal foresight, and impulsivity control [263]. The elevated risk of ADHD in children with OCD is of particular clinical concern due to the compounded impact of comorbidity. Children diagnosed with both disorders tend to exhibit an earlier onset of OCS, more severe functional impairment, and poorer academic outcomes compared to those with OCD alone. Moreover, they display a higher likelihood of comorbid conditions, including MDD, tic disorders, and disruptive behavior disorders. Importantly, treatment outcomes in this comorbid population are generally less favorable [266].

**Figure 2:**
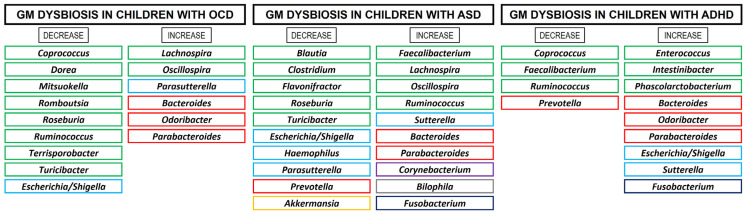
Comparative overview of GM dysbiosis in children with OCD, ASD, and ADHD. Rectangles in green: phylum Bacillota. Rectangles in light blue: phylum Pseudomonadota. Rectangles in red: phylum Bacteroidota. Rectangles in violet: phylum Actinomycetota. Rectangles in gray: phylum Thermodesulfobacteriota. Rectangles in yellow: phylum Verrucomicrobiota. Rectangles in dark blue: phylum Fusobacteriota.

Although OCD shares symptomatic features with both ASD and ADHD, it remains unclear whether these conditions also exhibit similar patterns of GM dysbiosis. Current evidence on this topic is still emerging, particularly in pediatric populations. Figure 2 presents a comparative overview of GM dysbiosis reported in children with OCD, ASD, and ADHD, based on findings from recent studies [223,267,268,269,270,271,272,273,274].

The genus *Bacteroides* has consistently been reported as increased in abundance across children diagnosed with OCD, ASD, and ADHD. Species within this genus are among the most prevalent across the human GM and are major producers of the SCFA propionate [275]. Elevated fecal levels of propionate have been observed in children with ASD, and administration of propionic acid in rodent models has been shown to induce behavioral phenotypes resembling core features of ASD [276]. Beyond SCFA production, *Bacteroides* also participates in the metabolism of tryptophan, yielding indole and other bioactive metabolites in the gastrointestinal tract. These microbial-derived tryptophan metabolites have been implicated in GBA signaling through their capacity to modulate immune function and activate neural pathways [277].

Another genus reported as increased in both OCD and ASD is *Lachnospira*, a butyrate-producing taxon. However, findings regarding its role in neurodevelopmental and neuropsychiatric disorders remain inconsistent. While some studies report elevated levels of *Lachnospira* in OCD and ASD, a reduction in its abundance has been observed in individuals with GAD [278]. Although anxiety is a distinct diagnostic category, it frequently co-occurs with both OCD and ASD, raising the possibility of shared GM-mediated pathways. Butyrate, the primary SCFA produced by *Lachnospira*, has been shown to exert diverse physiological effects, including the regulation of cell signaling pathways, modulation of neurotransmitter systems, attenuation of oxidative stress, and immune system modulation [276,279]. These mechanisms support the hypothesis that alterations in *Lachnospira* abundance may influence brain function via GBA interactions.

Increased abundance of the genus *Parabacteroides* has also been documented in children diagnosed with OCD, ASD, and ADHD. This finding aligns with previous reports indicating elevated *Parabacteroides* levels in individuals with depression compared to healthy controls [280,281]. Despite its association with mood disorders, *Parabacteroides* has been linked to health-promoting properties, including SCFA production [282]. It has been proposed that its elevated abundance in depression may represent a compensatory microbial response to host dysregulation [283]. Notably, *Parabacteroides* species have been linked to the synthesis of γ-aminobutyric acid (GABA) and indole metabolites, both of which play pivotal roles in neural signaling. Disruptions in these metabolic pathways have been associated with affective disorders, including depression and anxiety [284], which underscores the potential role of this genus in neuropsychiatric conditions.

Conversely, a reduction in the abundance of the genus *Ruminococcus* has been reported in children diagnosed with OCD and ADHD. One proposed hypothesis suggests that this decrease may be functionally linked to the involvement of *Ruminococcus* in arginine metabolism. Specifically, reduced levels of *Ruminococcus* may lead to elevated circulating arginine concentrations, which could subsequently enhance nitric oxide production, a compound that, at high levels, has been proposed to exert neurotoxic effects [285]. In addition to *Ruminococcus*, decreased relative abundances of several other common bacterial genera have been observed in these populations, including *Dorea*, *Roseburia*, and *Turicibacter*.

## 6. Psychobiotics as Therapeutic Tools for OCD

To date, the first-line treatments for OCD include psychotherapy, particularly cognitive–behavioral therapy (CBT) and pharmacotherapy with SSRIs [286,287]. In exposure and response prevention CBT, patients are gradually encouraged to confront their obsessive thoughts while resisting the urge to engage in compulsive behaviors. This process helps reduce anxiety associated with obsessions and weakens the connection between obsessions and compulsions. Pharmacologically, SSRIs such as fluoxetine, sertraline, fluvoxamine, paroxetine, and escitalopram work by increasing serotonin levels in the brain, either alone or in combination with glutamate modulators, to alleviate OCS [288]. However, these treatments have significant limitations, so exploration of new therapeutic approaches has recently begun, including deep brain stimulation, repetitive transcranial magnetic stimulation, and the use of psychobiotics [232,286,289,290].

The term psychobiotic was coined by Dinan et al. [291] to describe a new class of psychotropics defined as a living organism that, when ingested in adequate amounts, produces a health benefit in patients suffering from psychiatric illnesses. Since then, this definition has been expanded to include any exogenous influence whose effect on the brain is mediated by bacteria [292]. Thus, psychobiotics contain microorganisms and/or substances that affect the signaling of the GBA, such as probiotics, prebiotics, synbiotics, postbiotics, and even plant-derived compounds [293], and they may be delivered via supplements, functional foods, nutraceuticals, or dietary intake enhancements [294].

Probiotic and prebiotic interventions support the possible role of the GM on OCD symptomatology in preclinical studies. Treatment with *Lacticaseibacillus* (formerly *Lactobacillus*) *rhamnosus* strain GG attenuated the experimental induction of OCS in mice [32]. The administration of the probiotic *Limosilactobacillus* (formerly *Lactobacillus*) *reuteri* significantly reduced repetitive behaviors in mice [295]. Another study conducted in murine models demonstrated the efficacy of *Lacticaseibacillus casei* strain Shirota in the treatment of OCD through modulation of serotonin-related genes [34]. Simultaneous treatment of the probiotic with fluoxetine increased BDNF expression, while serotonin receptor 2A expression decreased in the orbitofrontal cortex [34]. In the case of prebiotics, a study in animal models found that mice receiving a prebiotic mixture composed of short-chain galactooligosaccharides and long-chain fructooligosaccharides exhibited changes in the serotonergic system [296]. In the brains of the treated group of mice, altered mRNA expressions of astrocytic glial fibrillary acidic protein and microglial integrin alpha-M (or Mac-1) were observed. In addition, increased BDNF mRNA expression was also noted in the prefrontal cortex [296]. Treatment with the synbiotic consisting of bee pollen (as a prebiotic agent) and the probiotic cocktail ProtexinR, which contains *Bifidobacterium longum* subsp. *infantis*, *Bifidobacterium breve*, *Lacticaseibacillus acidophilus*, *Lactobacillus delbrueckii* subsp. *bulgaricus*, *L. casei*, *Lacticaseibacillus rhamnosus*, and *Streptococcus thermophilus*, reversed the effects of propionic acid on cognitive dysfunction in rodents [297].

Psychobiotic treatments have also been conducted in humans to alleviate OCS. Patients treated with a probiotic formulation containing *Lacticaseibacillus helveticus* strain R0052 and *Bifidobacterium longum* strain R0175 reported decreased anxiety and stress symptoms [298], and administration of only the *B. longum* strain improved scores on the OCS severity index. In a single case report, administration of the yeast *Saccharomyces boulardii* was found to reduce typical OCD behaviors [299]. Table 2 presents various preclinical and clinical studies on the effects of psychobiotic treatments on OCS.

## 7. Discussion

The present review aimed to examine current evidence on the etiology of childhood-onset OCD, with a particular focus on the involvement of GM alterations in the mechanisms related to the pathophysiology of the disorder. As a result of the analysis conducted, several key points should be noted: (i) the genetic factors of OCD are very complex and, despite advances in genomic techniques such as GWAS and WES, identifying specific risk loci remains challenging due to the polygenic and heterogeneous nature of the disorder; (ii) limited genetic findings in OCD suggest that epigenetic mechanisms, including DNAm and miRNA, play a role in mediating gene–environment interactions and influencing disorder risk; (iii) CSTC circuit abnormalities are central to OCD pathophysiology and OCS seem to arise from multifaceted dysregulation rather than from a single neural locus; (iv) limbic structures such as the amygdala play a pivotal role in emotional regulation in OCD, underscoring the importance of circuit-level approaches; (v) key neurotransmitters within CSTC circuits, such as dopamine, glutamate, and serotonin, contribute OCD pathophysiology, with evolving evidence highlighting glutamatergic dysfunction; (vi) evidence supporting PANDAS and PANS suggests that infections and autoimmune reactions can trigger or exacerbate OCS, particularly in pediatric populations; (vii) the psychological mechanisms underlying OCS are characterized by an interplay of cognitive and behavioral processes that contribute to the onset and persistence of the disorder; (viii) ACEs contribute to OCD onset through cognitive and emotional processes, including the self-conscious emotions of humiliation and shame; (ix) OCD symptomatology may be influenced by GM alterations, particularly in relation to inflammation and neuroimmune interactions; (x) PANDAS show convergent OCS and altered GM profiles, suggesting a microbiota-immune-brain axis involvement; (xi) human studies report reduced bacterial diversity and lower levels of butyrate-producing genera in OCD, which may compromise gut integrity and contribute to neuroinflammation and dopaminergic dysregulation; (xii) specific microbial taxa may confer either protective or risk-modifying effects for OCD, supporting a potential causal role of GM composition; (xiii) immune markers such as altered IgA levels and increased susceptibility to infection in OCD populations point toward a dysfunctional GM-immune system relationship; (xiv) alterations in GM composition can modulate the synthesis and regulation of key neurotransmitters involved in OCD pathophysiology, including dopamine, glutamate, and serotonin; (xv) GM dysbiosis may reduce tryptophan availability, compromising central serotonin synthesis and potentially affecting BBB integrity; (xvi) specific bacterial taxa have been shown to influence dopaminergic and glutamatergic pathways, suggesting a functional link between microbial balance and neural circuits underlying OCD; (xvii) preclinical and clinical studies indicate that GM shifts are associated with changes in neurotransmitter receptor and transporter expression, which may contribute to anxiety, compulsivity, and impaired inhibitory control; (xviii) disruptions in the GM during early life may impair neurotransmitter synthesis, nerve myelination, and gastrointestinal health, thereby increasing the risk for neurodevelopmental and psychiatric disorders such as OCD; (xix) GM dysbiosis patterns show convergence across OCD, ASD, and ADHD, characterized by increased abundance of *Bacteroides*, *Lachnospira*, and *Parabacteroides*, alongside decreased levels of *Ruminococcus*, which highlights the GBA as a promising target for understanding and treating neurodevelopmental and psychiatric comorbidities in the context of OCD; (xx) preclinical studies have demonstrated that specific probiotic strains, such as *L. rhamnosus*, *L. reuteri*, and *L. casei*, reduce compulsive-like behaviors and modulate serotonergic gene expression; and (xxi) clinical evidence indicates that psychobiotic supplementation may reduce anxiety, stress, and OCS, representing a promising adjunctive therapeutic approach for OCD.

Based on the review conducted, it can be asserted that OCD constitutes a mental health condition with a complex, multifactorial etiology involving genetic, neural, immunological, psychosocial, and microbial factors that underlie its characteristic compulsivity and impairments in cognitive control and flexibility. However, the mechanisms implicated in the onset and development of OCD remain incompletely understood. Individuals with OCD spend a substantial amount of time and effort alleviating the stress generated by their obsessions through compulsive behaviors, aiming to prevent anticipated negative outcomes. These psychological processes are often ineffective, maladaptive, and even counterproductive. For instance, individuals with an intense fear of germ-related elements avoid contact with certain environments, believing they are protecting their health [301], but they may inadvertently compromise their immune system [185]. Despite its apparent dysfunctional essence, evolutionary theories of OCD highlight that certain compulsive behaviors may have conferred adaptive advantages in ancestral environments, suggesting that the interaction between inherited predispositions and current social contexts influences the expression and maintenance of OCS [302]. Extending this perspective to contemporary contexts, it is plausible that certain OCD-related traits continue to confer selective benefits. For instance, heightened attention to detail, risk aversion, and repetitive checking behaviors may enhance safety, accuracy, and task performance in specific occupational or environmental settings. In fact, these traits could promote meticulousness and thoroughness, qualities advantageous in professions such as scientific research, editorial work, or quality control. This underscores the potential value that individuals with mental health conditions can offer to society, highlighting the need to counteract the prevailing social stigma that frequently labels these individuals as burdens. In this respect, promoting appropriate social integration and support mechanisms is essential to capitalize on their strengths and improve both personal and societal outcomes. Nevertheless, it is important to acknowledge that some individuals who are capable of contributing to society may overstate the severity of their mental health condition in order to obtain accommodations. This phenomenon presents a challenge that must be addressed through diagnostic assessments that accurately determine the functional capacity of affected populations, thereby informing effective treatment strategies and ensuring the equitable allocation of healthcare resources. In light of these considerations, advancing our understanding of the underlying mechanisms of mental disorders is pivotal, not only to refine clinical approaches, but also to inform evidence-based policies.

OCD involves significant complications arising from the broad variability in symptom presentation and frequent comorbidities [77]. The clinical and etiological heterogeneity of OCD poses challenges for both research and clinical management [303,304]. While classifying OCD into discrete subtypes has provided insights, consistent biological markers and reliable predictors of treatment response remain to be fully elucidated. These challenges point to the utility of dimensional models that characterize the disorder as a spectrum of co-occurring symptom dimensions instead of discrete, mutually exclusive subtypes, potentially facilitating the identification of both common and specific etiological mechanisms underlying each symptom dimension [305].

The conceptualization, clinical definition, and treatment outcomes of OCS have been previously debated, reflecting uncertainty in both phenomenological and therapeutic research [306,307,308,309]. Therefore, identifying etiological factors and reliable predictors of treatment response is of paramount importance. Not Just Right Experiences (NJREs) constitute an endophenotypic marker observed across clinical and subclinical populations as well as unaffected relatives, which reflects familial vulnerability [310]. Neurobiological evidence implicates altered sensory-motor integration and overactive performance monitoring as potential mechanisms underlying NJREs [311,312,313]. Notably, overactive performance monitoring has been shown to persist in adult OCD patients even after successful symptom reduction through CBT, highlighting its role as a stable vulnerability marker rather than a state-dependent symptom [311]. From a psychosocial perspective, NJREs provide a conceptual bridge linking sensory-perceptual abnormalities to emotional regulation deficits, potentially contributing to vulnerability via both genetic and environmental pathways.

The impact of GM dysbiosis on various mental health disorders has been previously demonstrated [28]. However, its involvement in OCD is still under investigation, mainly due to the multifaceted nature of this disorder regarding its etiology and development, and also because the GM can influence genetic, immunological, and neuronal aspects that may be associated with the pathophysiology of OCD [314]. Although there are very few studies on the relationship between the GM and OCD, several authors have concluded that GM dysbiosis induces an inflammatory process in OCD patients due to the synthesis of microbial metabolites, such as trimethylamine N-oxide, indole derivatives, lipopolysaccharides, SCFAs, bile acids, and N-phenylacetyl-L-glutamine [315,316].

The comorbidity of various mental disorders with OCD has been described in several studies. In this regard, specific conditions such as MDD, GAD, or bipolar disorder share the involvement in inflammatory and neuroinflammatory processes [220,317]. These inflammatory processes are more prevalent in patients with OCD who present gastrointestinal disturbances [318]. However, a clear relationship between the clinical manifestations of OCD and GM dysbiosis cannot be conclusively established, so it is pivotal to study the specific gut microorganisms that could trigger the development of OCD, or to investigate disease biomarkers in order to select appropriate psychobiotics as adjuvant therapy.

Clinical trials investigating the effects of psychobiotics across various mental disorders [293], including OCD [226], have reported contradictory results. One of the main reasons for this disparity in results, as well as the fundamental challenge for future studies, is that, unlike animal models, interventions applied in humans present significant heterogeneity with respect to diet, age range, genetic characteristics, and GM composition [319,320]. This variability at the microbial colonization level may contribute to various effects, essentially those exerted by probiotics, both on the hosts themselves and on their GMs [320]. This is understandable, since the microorganisms that are part of the GM can influence the growth of probiotics, so if there is a previous dysbiosis, the effects of the probiotic intervention in the host can be altered. Another alternative treatment to psychobiotics is fecal microbiota transplantation (FMT), which is an option with less variability in outcomes by enabling the transplantation of an entire microbial community [232]. However, the main drawback is that there is currently insufficient data available to monitor the potential risks to which patients are exposed when they are transplanted with the fecal microbiota of another healthy individual. Despite this, FMT constitutes a very promising treatment approach, as it has demonstrated satisfactory results for the treatment of several mental health conditions and gastrointestinal diseases [321,322].

In recent years, the field of nutritional psychiatry has gained growing influence in clinical settings, highlighting the importance of rigorous scientific approaches to evaluate the efficacy and appropriate use of dietary interventions. This discipline increasingly advocates for the integration of objective biomarkers, including inflammatory cytokine levels, nutrient deficiencies, genomic and microbiome profiles, and dietary patterns, in assessing the interplay between nutrition and mental health [323]. Given that low intake of vegetables has been associated with a worsening of OCS over time, while diets high in saturated fats appear to correlate with increased compulsive behaviors [206], it is plausible to consider the potential value of plant-based dietary patterns combined with psychobiotic supplementation as a non-pharmacological strategy for alleviating OCS. Although research exploring the mental health effects of vegetarian diets has yielded mixed results due to methodological limitations and several confounding factors [324], these diets may offer significant therapeutic benefits through the consumption of metabolic derivatives such as SCFAs and polyamines, which have been linked to improvements in cognitive decline and mood-related mental health conditions [319].

OCD imposes significant cognitive, emotional, and functional burdens on those affected, leading to a marked reduction in their quality of life [325]. In this sense, individuals with OCD often exist in a permanent state of distress, with OCD consuming substantial amounts of time and energy, which hinders productivity and personal performance. In social terms, OCD is linked to isolation and loneliness [326,327], as individuals with the disorder tend to avoid situations that trigger their aversive symptoms, and may also feel ashamed of their own obsessions and compulsive behaviors. The stigma associated with OCD further exacerbates these difficulties, as misconceptions about the disorder lead to prejudice and social discrimination, obliterating the willingness of individuals to seek support [328]. Moreover, in recent times, the COVID-19 pandemic has led to public health campaigns portraying microbes as harmful, resulting in the widespread fear of interpersonal contact and adoption of non-specific sterilization practices in everyday settings [329]. These initiatives, along with global prevention messages and transmission control measures, have significantly impacted the prevalence of OCD and the quality of life of those affected [330]. Indeed, current research has documented a significant rise in OCS during and after the COVID-19 pandemic, with variations in prevalence and in severity across different populations [331,332,333,334], but especially affecting vulnerable demographic groups, particularly children and adolescents [335,336]. Specifically, public health messaging and the associated responsibility to prevent harm appeared to worsen contamination-related OCS [337], exacerbating stress-related anxiety responses [333,338], which underscores the need for innovative and customized therapeutic strategies, such as tele-therapy [339] and e-mental health resources [340].

In the context of this discussion, it is worth noting that oral health, as an integral component of systemic homeostasis, may also intersect with the etiological pathways of OCD. Oral health represents a vital aspect of overall well-being, and emerging evidence suggests that alterations in the oral microbiome may contribute to the onset and development of certain mental health conditions through systemic inflammation, neuroimmune signaling, and microbiota-GBA interactions [341]. A recent cross-sectional study conducted by Martinovic et al. [342] found that individuals exhibiting higher obsessive–compulsive personality disorder tendencies (e.g., perfectionism, fastidiousness, punctiliousness) demonstrated significantly better oral hygiene practices compared to peers with lower tendencies. In addition, the study acknowledges the potential risks of excessive behavioral rigidity, such as over-brushing, which could adversely affect dental health. Taken together, these insights suggest that both the composition of the oral microbiome and the behavioral patterns modulating it may represent overlooked factors in the etiology of OCD.

Important limitations in the current epidemiological evidence for OCD need to be acknowledged. Firstly, many studies rely on lay interviewer diagnoses of OCD. However, questions remain regarding the validity of this approach. Further research is needed to assess the concordance between lay interviewer diagnoses and clinical assessments. In addition, widely used survey tools in epidemiological research, such as the Composite International Diagnostic Interview, have not rigorously differentiated OCD from other related disorders, including body dysmorphic disorder, hoarding disorder, and Tourette syndrome. This convergence may lead to misclassification, either by diagnosing individuals with distinct disorders as having OCD or by failing to identify these disorders altogether. Ongoing and future research holds promise for refining global prevalence estimates and correlates of OCD.

Despite advances in understanding childhood-onset OCD, significant gaps remain in examining its determinants. Longitudinal studies are limited, hindering causal inferences between biological, microbial, and psychosocial factors. The characterization of the GM in pediatric OCD populations remains incomplete, and mechanisms linking GM alterations to neurobiological dysfunction require further elucidation. In addition, clinical trials investigating psychobiotic interventions are scarce, limiting evidence on therapeutic efficacy. Finally, an integrated multidisciplinary approach combining biological and psychosocial determinants is still lacking, constraining a comprehensive understanding of OCD development. Therefore, progressively integrating these domains is crucial for advancing the understanding and management of complex mental health conditions such as OCD.

Lastly, it should be noted that this narrative review presents various limitations that must be considered when interpreting its findings: (i) the heterogeneity of the included studies regarding methodologies and populations; (ii) variability in interventions and inconsistent outcomes measures across studies; (iii) no formal assessment of study quality or risk of bias was conducted due to the descriptive nature of this review; (iv) potential publication bias; and (v) limited longitudinal data available, which restricts conclusions about causality and temporal relationships.

## 8. Conclusions

OCD is a complex mental health disorder with its etiology influenced by the interplay of multiple determinants, including genetic and epigenetic mechanisms, brain function, the immune system, psychosocial factors, and GM alterations. OCD has a strong hereditary component, involving both common polygenic variants and rare mutations. However, the high heritability of OCD contrasts with limited GWAS findings, emphasizing the role of epigenetic factors in its complex etiology. Dysfunction and hyperactivity within CSTC circuits underlie one of the neurobiological bases of OCD, contributing to cognitive rigidity and compulsive behaviors through disrupted excitatory-inhibitory balance and impaired circuit integration. The involvement of multiple neurotransmitter systems in OCD suggests that effective treatment may require targeting beyond serotonin, incorporating dopaminergic and glutamatergic pathways. Immune system dysregulation may also contribute to OCD pathophysiology, although findings are inconsistent and confounded by secondary OCD cases. ELS plays a role in triggering the onset of OCD and shaping the clinical presentation of its symptoms. Moreover, psychosocial factors influence the severity and progression of OCD. OCS are maintained by maladaptive cognitive processes that amplify distress and compulsive behaviors, while impairments in cognitive flexibility and memory further interfere with symptom improvement.

Research on the relationship between OCD and the GM is still in its early stages. Emerging evidence suggests that GM dysbiosis may contribute to OCD pathophysiology through immune and neuroinflammatory mechanisms, particularly in pediatric populations. Although causality has yet to be firmly established, alterations in butyrate-producing bacteria and immune markers such as IgA support the relevance of the GBA in OCD onset and progression. In addition, neurotransmitter dysregulation in OCD may be influenced by GM alterations. In this respect, dysbiosis can affect neurotransmitter synthesis, receptor expression, and BBB integrity, thereby contributing to OCD neurobiology. In turn, environmental influences can disrupt GM development during critical neurodevelopmental periods, potentially contributing to long-term alterations in brain function and increasing vulnerability to psychiatric disorders, including OCD. There are similar GM dysbiosis patterns across OCD, ASD, and ADHD, involving increased abundance in *Bacteroides*, *Lachnospira*, and *Parabacteroides*, as well as reduced abundance of *Ruminococcus*, which suggests a contribution of GBA alterations to shared neurodevelopmental and neuropsychiatric mechanisms. Current first-line OCD treatments, including CBT and SSRIs, show efficacy but present notable limitations. Preclinical and clinical evidence indicated that psychobiotics may alleviate OCS by influencing serotonergic pathways, neurotrophic factors, and neuroinflammation.

Further research is needed to optimize treatment methods and to evaluate the efficacy and safety of microbial approaches in OCD. Additional human studies will provide insights to implement more customized and effective therapies for individuals with OCD, such as the development and application of probiotic cocktails or the use of OCD-specific synbiotics and postbiotics. These advancements will position microbiome therapeutics as an essential component of precision medicine within the context of mental health. Given the substantial burden that OCD imposes on individuals and the limited applicability and effectiveness of current frameworks and treatments, it is imperative to intensify research efforts exploring the role of the GM in the pathophysiology of OCD. In this respect, research based on GM approaches could provide new insights into the pathogenesis of the disorder and facilitate the development of innovative therapeutic strategies offering improved outcomes for those affected. In summary, it is plausible to state that the GM plays a pivotal role in OCD, constituting a promising approach for understanding the etiology of the disorder and highlighting the significant clinical potential of treatments based on the administration of psychobiotics.

## Figures and Tables

**Table 1:** Relationship between GM dysbiosis and OCD in preclinical and clinical studies.

References	Sample	Decrease	Increase
Preclinical			
Jung et al. [217]	Long-Evans rats treated with quinpirol (*N* = 15).	NE	*Lachnospiraceae* *Ruminococcaceae*
Scheepers et al. [218]	*Apodemus sylvaticus* mice (*N* = 11).	*Anaeroplasma* *Prevotella*	*Aestuariispira* *Desulfovermiculus* *Holdemanella* *Peptococcus*
Clinical			
Quagliariello et al. [219]	OCD patients with PANS/PANDAS (*N* = 30).	*Coprococcus* *Dorea* *Roseburia* *Turicibacter*	*Bacteroides* *Odoribacter* *Oscillospira*
Turna et al. [220]	OCD patients (*N* = 21).	*Anaerostipes* *Odoribacter* *Oscillospira*	NE
Domènech et al. [221]	OCD patients (*N* = 38).	*Agathobacter* *Coprococcus* *Prevotellaceae*	*Alistipes*
D’Addario et al. [222]	OCD patients (*N* = 64)	Fusobacteriota/Actiomycetota ratio	*Actinobacter* Bacteroidota
Dai et al. [223]	OCD children (*N* = 49)	*Escherichia* *Shigella* *Romboutsia* *Subdoligranulum* *Terrisporobacter* *Mitsuokella* *Coprococcus* *Ruminococcus*	*Bacteroides* *Lachnospira* *Parabacteroides* *Parasutterella*

NE: not specified.

**Table 2:** Effects of psychobiotics on OCS in preclinical and clinical studies.

References	Studies	Treatment	Outcomes
Preclinical			
Kantak et al. [32]	BALB/cJ mice (*N* = 12).	Probiotic: *L. rhamnosus* strain GG for 2–4 weeks.	Attenuates OCD-like behaviors (increased perseverative locomotion in the open field, stereotypic movements, and marble burying).
Tabouy et al. [295]	Shank3 KO mice (*N* = 31).	Probiotic: *L. reuteri* strain MM4-1A for 3 weeks.	Reduces repetitive and antisocial behaviors and GABA receptor expression.
Sanikhani et al. [34]	Wistar rats (*N* = 30).	Probiotic: *L. casei* strain Shirota *+* Fluoxetine for 4 weeks.	Modulates genes related to serotonin. Exerts an increase in BDNF.
Ghuge et al. [300]	Wistar rats (*N* = 30) treated with quinpirole	Probiotic cocktail: *Bacillus coagulans* strain Unique IS-2, *L. rhamnosus* strain UBLR-58, *L. plantarum* strain UBLP-40, *B. lactis* strain UBBLa-70, *B. infantis* strain UBBI-01, and *B. breve* strainUBBr-01 for 8 weeks	Probiotic cocktail reverses quinpirole-induced OCD-like phenotypes. Restores the dysregulated inflammatory mediators and neurotransmitters
Szklany et al. [296]	BALB/cByJ mice (*N* = 10).	Prebiotic: GOS + FOS for 11 weeks.	Reduces anxiety and improves social behaviors. Exerts changes in the serotonergic system.
Alghamdi et al. [297]	Wistar rats (*N* = 36).	Synbiotic: probiotic ProtexinR (*B. longum* subsp. *infantis*, *B. breve*, *L. acidophilus*, *L. delbrueckii* subsp. *bulgaricus*, *L. casei*, *L. rhamnosus*, and *Streptococcus thermophilus*) + prebiotic (bee pollen) for 3 weeks.	Reverses the effects of propionic acid on cognitive dysfunction. Protects from the neurotoxic effects of propionic acid.
Clinical			
Messaoudi et al. [298]	Healthy patients (*N* = 25) and OCD patients (*N* = 3).	Probiotic: *L. helveticus* strain R0052 and *B. longum* strain R0175 for 4 weeks.	Reduces anxiety and depression. Decreases the symptom severity index (Hopkins checklist).
Kobliner et al. [299]	A patient with OCD, ASD, and self-injurious behavior (*N* = 1).	Probiotic: *Saccharomyces boulardii.* The duration of treatment is not established.	Reduces symptoms related to OCD and self-injurious behaviors.

## Abbreviations

The following abbreviations are used in this manuscript:

OCD	Obsessive–compulsive disorder
OCS	Obsessive–compulsive symptoms
GM	Gut microbiome
GBA	Gut–brain axis
GAD	Generalized anxiety disorder
MDD	Major depressive disorder
ADHD	Attention-deficit/hyperactivity disorder
ELS	Early-life stress
CSTC	Cortico-striato-thalamo-cortical
GWAS	Genome-wide association studies
MAF	Minor allele frequency
CNV	Copy number variant
WES	Whole exome sequencing
DNAm	DNA methylation
miRNA	microRNA
EWAS	Epigenome-wide association studies
fMRI	Functional magnetic resonance imaging
SRIs	Serotonin reuptake inhibitors
SSRIs	Selective serotonin reuptake inhibitors
PANDAS	Pediatric autoimmune neuropsychiatric disorder associated with streptococcal infection
PANS	Pediatric acute-onset neuropsychiatric syndrome
TAF	Thought–action fusion
NP	Negative priming
IBA	Inverse Bayesian account
SLEs	Stressful life events
ACEs	Adverse childhood experiences
SCFA	Short-chain fatty acid
CNS	Central nervous system
MR	Mendelian randomization
BBB	Blood–brain barrier
ASD	Autism spectrum disorder
GABA	γ-aminobutyric acid
CBT	Cognitive–behavioral therapy
GOS	Galactooligosaccharides
FOS	Fructooligosaccharides
NJREs	Not just right experiences
FMT	Fecal microbiota transplantation
